# Engineering synthetic antibody binders for allosteric inhibition of prolactin receptor signaling

**DOI:** 10.1186/s12964-014-0080-8

**Published:** 2015-01-15

**Authors:** Shahir S Rizk, Jean-Louis K Kouadio, Anna Szymborska, Erica M Duguid, Somnath Mukherjee, Jiamao Zheng, Charles V Clevenger, Anthony A Kossiakoff

**Affiliations:** Department of Biochemistry and Molecular Biology, The University of Chicago, Chicago, IL USA; Current Address: Boler –Parseghian Center for Rare and Neglected Diseases, University of Notre Dame, Notre Dame, IN USA; Current Address: Monsanto Co. 700 Chesterfield Parkway, Chesterfield, MO USA; Current Address: Max Delbrueck Center for Molecular Medicine, Berlin, Germany; Department of Pathology, Northwestern University, Chicago, IL USA; Current Address: Department of Pathology, Virginia Commonwealth University, Richmond, VA USA

**Keywords:** Prolactin signaling, Synthetic antibody, Phage display, Allostery

## Abstract

**Background:**

Many receptors function by binding to multiple ligands, each eliciting a distinct biological output. The extracellular domain of the human prolactin receptor (hPRL-R) uses an identical epitope to bind to both prolactin (hPRL) and growth hormone (hGH), yet little is known about how each hormone binding event triggers the appropriate response.

**Findings:**

Here, we utilized a phage display library to generate synthetic antibodies (sABs) that preferentially modulate hPRL-R function in a hormone-dependent fashion. We determined the crystal structure of a sAB-hPRL-R complex, which revealed a novel allosteric mechanism of antagonism, whereby the sAB traps the receptor in a conformation more suitable for hGH binding than hPRL. This was validated by examining the effect of the sABs on hormone internalization via the hPRL-R and its downstream signaling pathway.

**Conclusions:**

The findings suggest that subtle structural changes in the extracellular domain of hPRL-R induced by each hormone determine the biological output triggered by hormone binding. We conclude that sABs generated by phage display selection can detect these subtle structural differences, and therefore can be used to dissect the structural basis of receptor-ligand specificity.

**Electronic supplementary material:**

The online version of this article (doi:10.1186/s12964-014-0080-8) contains supplementary material, which is available to authorized users.

## Findings

It is commonplace for a single receptor to recognize a number of different ligands and a single ligand to bind to a number of different receptors [1]. In many cases, the distinct biological output elicited by each ligand is due to its interaction with a different epitope on the surface of a receptor, triggering a defined signaling pathway. In some unique cases, a receptor is able to interact with different ligands using an identical set of amino acids, yet each ligand results in a distinct biological outcome.

A prominent example of this type of promiscuity is the prolactin receptor (PRL-R), which uses the same set of amino acids on its extracellular domain (ECD) to bind to two different hormones: prolactin (PRL) [2,3] and growth hormone (GH) [4,5]. Prolactin receptor signaling is primarily triggered by homodimerization [3], which is induced by GH or PRL binding. Since the structures of hGH and PRL contain no tertiary symmetry, in forming their respective complexes with the ECD homodimers, the hormones employ opposite faces utilizing surface epitopes possessing quite different topographical and electrostatic features [4-6]. As a consequence, in the case for both PRL and GH, the ECD binding sites have quite different affinities: a high affinity site called Site 1, and a lower affinity site, Site2 [6]. Although Site1 has higher affinity than Site2, quite remarkably, the paratope they interact with on the ECD is virtually identical. Thus, the same binding patch on the PRL-R ECD recognizes two distinct binding interfaces on each of two different hormones, yet each hormone activates a distinct biological outcome. Exactly how this is accomplished is an area of intense interest in both basic and applied research and many unresolved molecular recognition issues remain.

We present, here, a methodology that utilizes a novel class of affinity reagents generated by phage display selections that induce allosteric changes in a target protein that can effectively differentiate between ligands that bind to the same receptor epitope. These affinity reagents are based on the antigen-antibody fragment (Fab) antibody frameworks and are called “synthetic antibodies” or sABs [7]. The extracellular domain of the human prolactin receptor (hPRL-R) was chosen as a target system because of its number of activating hormones [5,6,8] and the biological implications of being able to selectively tune its activity [3,9,10]. Structural studies comparing the two hormone-receptor complexes [4] suggested that such selectivity could be achieved by capturing subtle conformational differences induced by hGH versus hPRL at either site in the ECD through an allosteric-control mechanism.

To generate sABs against hPRL-R, the immobilized ECD was incubated with a 10^10^ member reduced genetic code phage display library [11]. The sABs are displayed in bivalent format by fusion to the pIII coat protein of M13 phage. Diversity is introduced into four of the six complementary determining region (CDR) binding loops. After three rounds of selection followed by a binding screen by phage ELISA, four unique sAB clones were identified. All four sABs, designated A4, A8, A9 and A10, were expressed and purified, and their affinities for the hPRL-R ECD were determined by surface plasmon resonance (SPR) (Table 1). Three of the four sABs exhibited nanomolar affinities for the hPRL-R ECD. sAB, A4 showed little detectable binding in a protein format and thus was used as a negative control in the subsequent experiments.

**Table 1:** **Binding kinetics and loop sequence of PRL-R sABs**

**sAB**	**k**_**on**_ **(M** ^**−1**^ **s** ^**−1**^ **)** ^**a**^	**k**_**off**_ **(s** ^**−1**^ **)** ^**a**^	**K**_**D**_ **(nM)** ^**a**^	**Loop sequence**			
				**L3** ^**b**^	**H1** ^**b**^	**H2** ^**b**^	**H3** ^**b**^
**A-4**	No binding			HYTTPP	FYSSYI	YIYPSYGY	SYYSYYGWDYHNSSGAM
**A-8**	9.6 × 10^4^	5.4 × 10^−4^	5.6	YYSYYYPF	FSSSSM	YISPYYGS	SYGYVYWNAYSSGM
**A-9**	1.4 × 10^4^	3.4 × 10^−5^	2.5	YSSSYLL	FSSSSI	SIYSYYSS	SYDSYPWVYSYTVSGAF
**A-10**	1.2 × 10^5^	2.6 × 10^−3^	21	HYTTPP	FSSSYI	SISPYYGS	SYYYPEETAF

^**a**^Binding kinetics were determined by SPR on a Biacore 2000, dissociation constant was calculated from the on and off rates.

^**b**^Randomized loops in the Fab scaffold, L3: light chain loop 3; H1, H2 and H3: heavy chain loop 1, 2 and 3, respectively.

**Figure 1:**
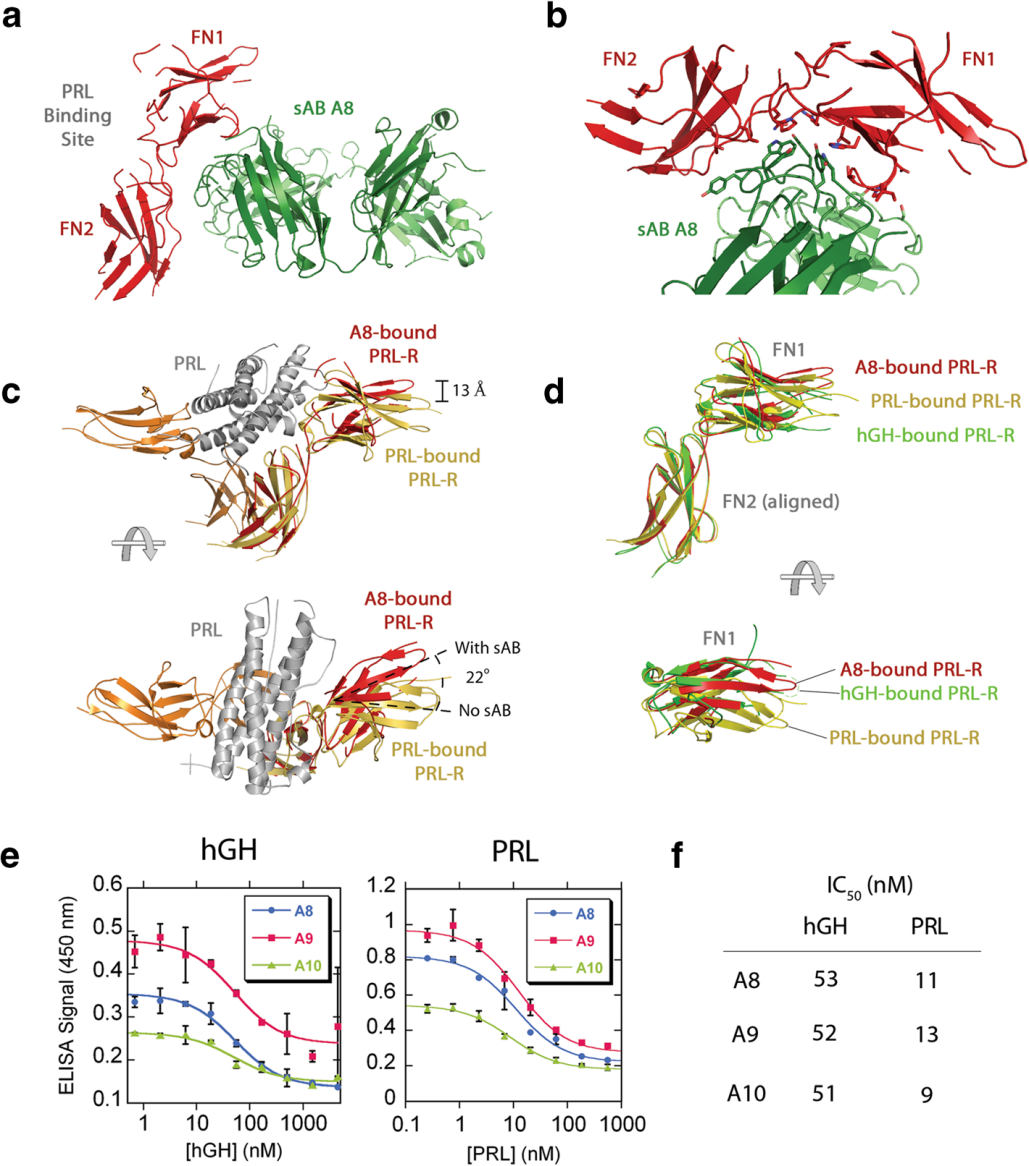
**Crystal structure of the hPRL-R in complex with sAB A8. a** sAB A8 (green) binds to the human PRL-R (red) opposite to the hormone binding site. **b** A close up of the interactions between the CDR loops of sAB A8 (green) and the hinge region between the two fibronectin (FN) domains of PRL-R (red). **c** Alignment of the FN2 domains of the PRL-R structure determined here (red) with the PRL-R structure (yellow) bound to PRL (grey) in the 2:1 complex (PDB code: 3NPZ), the side view (above) shows the twist between the two FN domains induced by sAB binding and the top view (below) shows a rotation of the FN1 domain relative to the FN2 domain in the sAB-bound structure. **d** Alignment of the sAB-bound PRL-R (red) with the hGH-bound PRL-R (green) and the PRL-bound PRL-R (yellow) using the FN2. The bottom panel is a top view of the FN1 of all three structures, with the sAB-bound receptor more resembling the hGH-bound conformation. **e** Inhibition of sAB binding by hGH or PRL using phage ELISA. Both hGH (left panel) and PRL (right panel) inhibit the interaction between phage-displayed sABs and hPRL-R in a concentration dependent manner. **f** IC_50_ values of the hGH or PRL inhibition of the interaction between sABs and hPRL-R calculated from **e**.

To gain insight into the structural basis of the interactions between the sABs and hPRL-R, we determined the crystal structure of the receptor in complex with sAB A8. In the structure, the sAB binds to the opposite face of the ECD away from the hormone-binding site (Figure 1a). Interactions between the two molecules are mediated by side chain residues of the CDR loops of the sAB and the hinge region that connects the two tandem fibronectin (FN) domains of hPRL-R (Figure 1b). Alignment of the ECD FN2 domain in the context of the sAB-bound structure with the 2:1 PRLR: PRL structure [8] reveals that the sAB captures a hinge-bending conformation that results in a subtle, but significant rotation of FN1 relative to FN2 (Figure 1c).

Interestingly, the receptor in complex with the sAB more closely resembles the hGH-bound form than the PRL-bound form [4] (Figure 1d). By aligning the FN1 domains of all three structures, the FN2 of the sAB-bound form and the hGH-bound form align well, while the FN2 of the PRL-bound is offset from the other 2 structures (Figure 1d). This observation implies that the sAB would have a greater impact on PRL binding than hGH binding. To test this, we examined the ability of each hormone to inhibit sAB binding to the receptor using a phage ELISA. In this experiment, the biotinylated receptor was immobilized on streptavidin plates and incubated with increasing concentrations of either hGH or PRL in the presence of phage displaying sAB A8, A9 or A10. By detecting the relative amount of phage bound to the receptor at each hormone concentration, IC_50_ values for each hormone-sAB combination were determined (Figure 1e). Although hGH has a higher affinity for the receptor (*K*_d_ ~1nM) [6] than PRL (*K*_d_ ~6nM) [2], the IC_50_ values for hGH were ~5 fold higher than those for PRL (Figure 1f). This indicates that PRL has a more profound inhibitory effect on sAB-receptor interaction than hGH, an observation that is consistent with structure alignments (Figure 1d). The results also suggest that all three sABs bind to the same epitope on the receptor, supported by basic epitope mapping using phage ELISA (Additional file 1: Figure S2).

**Figure 2:**
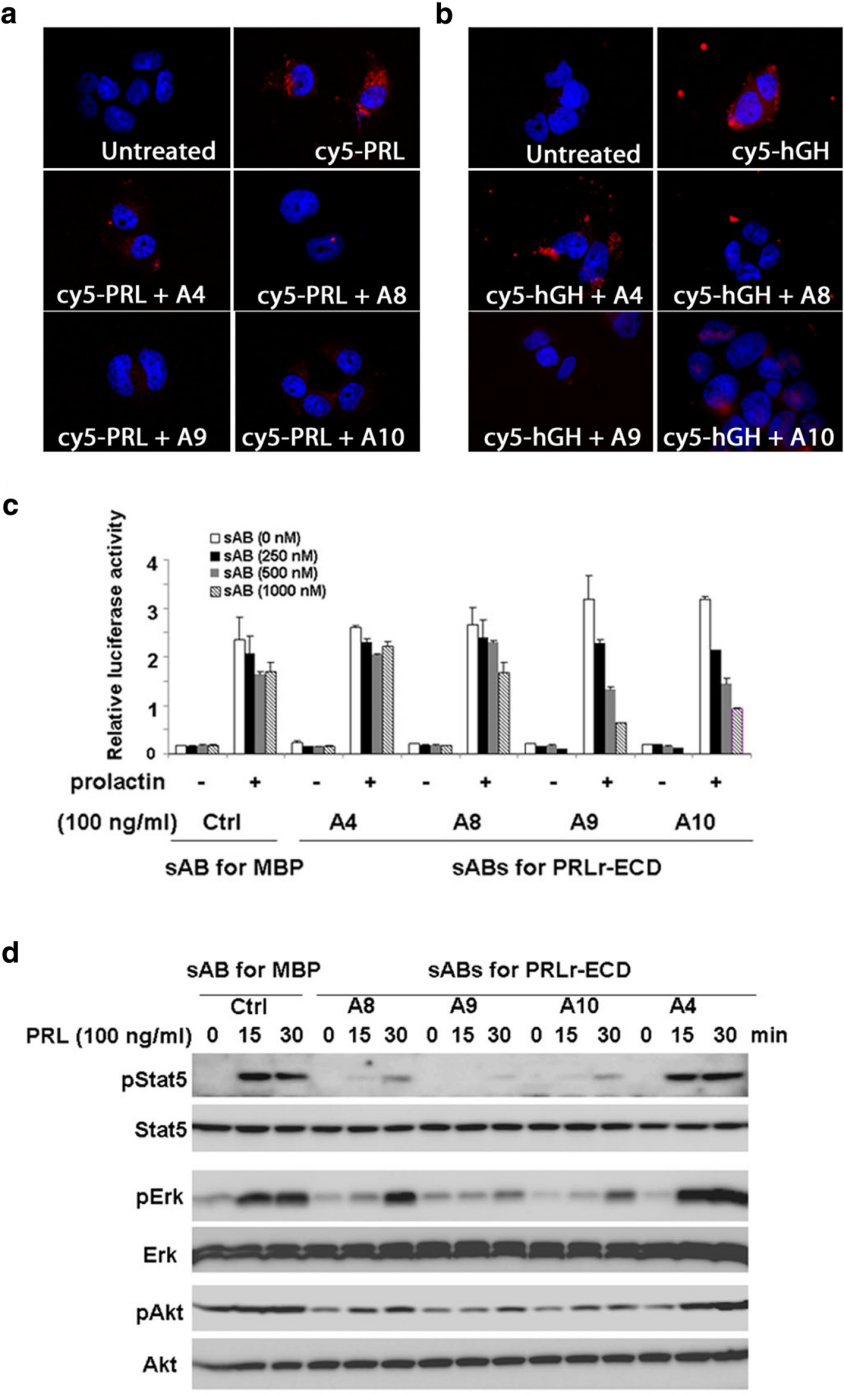
**sAB inhibition of hPRL-R in T47D breast cancer cells. a**-**b** Fluorescence microscopy images of cells treated with 100 nM cy5-hPRL **(a)** or cy5-hGH **(b)** in the presence of each sAB. All panels represent two merged channels; blue: DAPI nuclear stain, red: cy5. **c** A Stat5 phospholyration luciferase reporter assay of T47D cells in the presence or absence of hPRL and hPRL-R sABs. A sAB against bacterial maltose binding protein (MBP) [7] was used as a negative control. **d** Western blot detection of the time-dependent inhibition of hPRL-R signaling by hPRL-R sABs.

We note that biological signaling requires the hormones to bind the ECD at both Site1 and the lower affinity Site2. Thus, it is possible that the sABs have an even more profound effect on ECD binding at Site2. Unfortunately, because of its more transient nature, it is not possible to quantitatively measure the influence of sABs on binding of the hormone at Site2. Thus, we instead quantified the effects using relevant biological readouts of the intracellular signaling mechanism mediated by the hPRL-R using T47D, a breast cancer cell-line. First, we examined the effects of sABs on hormone-induced receptor internalization, which is a ubiquitous feature of cytokine receptor activation. We tested the ability of the four sABs to block hPRL-R-mediated internalization of cy5-labeled hPRL or hGH. Fluorescence microscopy indicated that sABs A8, A9 and A10 significantly decreased or completely abolished the internalization of hPRL, whereas the control sAB A4 had no effect on hormone internalization (Figure 2a). In contrast, internalization of hGH was only slightly inhibited by sABs A8, A9 and A10 (Figure 2b), presumably due to the ability of the sABs to recognize the conformation differences between the hPRL and hGH bound forms of hPRL-R.

We next examined the concentration and time-dependent effects of the sABs on hPRL-R signaling. A luciferase reporter assay [12] revealed that sABs A8, A9 and A10 exhibit a concentration-dependent inhibition of downstream Stat5 phosphorylation, whereas the control sAB A4 showed no significant effect (Figure 2c). The inhibition of hPRL-R signaling by these three sABs was confirmed by western blot analysis of the downstream components of the prolactin induced signaling pathway (Figure 2d). These sABs drastically inhibited Stat5 and ERK phosphorylation, and partially inhibited AKT phosphorylation. Additionally, inhibition of signaling is correlated with the affinity of each sAB for the receptor.

The structural information gained from the A8-hPRL-R complex reveals the mechanism by which the sAB functions as an allosteric antagonist of PRL activity. The ability of a number of cytokine receptors, including hPRL-R, to bind to multiple ligands, which manifest in different biological responses, brings about important questions relating subtle changes in receptor conformation on the cell-surface to downstream effects inside the cell. Our work indicates that sABs can distinguish between such subtle differences in receptor structure, *i.e.* hGH-bound vs. hPRL-bound, that ultimately define the function of the receptor. Therefore, the ability to utilize phage display to rationally engineer sophisticated reagents as highly selective inhibitors with defined behavior can serve as a powerful approach to investigate the diversity of structural states of cell-surface receptors and to design new drugs.

## Additional file

Additional file 1:
**Supplementary information.**

## Abbreviations

sAB: Synthetic antibodies
hPRL-R: Human prolactin receptor
hPRL: Human prolaction
hGH: Human growth hormone
SPR: Surface Plasmon Resonance
FN: Fibronectin
