# Supplementary material for: Engineering synthetic antibody binders for allosteric inhibition of prolactin receptor signaling
Source: Cell Commun Signal. 2015 Jan 15;13:1. doi: 10.1186/s12964-014-0080-8 (PMC4300558; doi:10.1186/s12964-014-0080-8)
Supplement: Additional file 1: — Supplementary information. [file 12964_2014_80_MOESM1_ESM.doc]

**Supplementary information**

**Protein Expression and Purification**

T207C hPRL-R extracellular domain was expressed as described [1]. Harvested cell pellets were lysed in 20 mM Tris-HCl, pH 8.0 with 0.2mg/mL lysozyme and DNAse I. The protein was purified by affinity chromatography on hGH-conjugated Sepharose resin. Running and washing buffers for purification of T207C hPRL-R were supplemented with 20μM ZnCl_2_. hPRL-R was eluted in 4.5M MgCl_2_ and dialyzed against phosphate buffered saline (PBS) pH 7.4. Protein concentrations were determined by absorbance measurements at 280 nm using a molar extinction coefficient of 66,140 M^-1^cm^-1^.

sABs were expressed in 55244 cells as described [2]. The sABs were purified by a gravity protein A column, eluted with 100 mM H_3_PO_4_, 50 mM NaH_2_PO_4_, 140 mM NaCl, pH 2.0, then immediately, the pH was adjusted to 8 using 1 M TRIS. The sABs were further purified by ion exchange chromatography on HiTrap™ SP resin using a 0 – 150 mM NaCl gradient over 100 mL.

**Selection for sABs against hPRL-R**

hPRL-R was incubated with 1.5 mM DTT for 30 min, then a NAP5 desalting column was used to remove the DTT. Biotinylation was carried out using a 10-fold molar excess of EZ-Link Biotin-HPDP (Thermo) in PBS pH 7.4, 1 mM EDTA for 1 hr at room temperature, followed by removal of excess biotinylation reagent using a NAP5 column. We utilized a randomized FAB library fused to the pIII coat protein with four randomized CDR loops with biased sequence identities for tyrosine, glycine and serine [3]. Three cycles of selection were carried out as described previously [2]. Briefly, biotinylated hPRL-R (0.2 nmols) immobilized on 200 μl of washed streptavidin magnetic beads (Promega) was exposed to 1 mL of phage library containing ~10^12^ unique clones. Unbound phage was washed and the beads containing the bound phage were used to infect 3 ml of log-phase XL1-Blue *E. coli* for 20 minutes at room temperature. The mixture was then transferred to 30 ml of 2xYT media supplemented with ampicillin (100μg/ml) and approximately 10^8^/ml of M13K07 helper phage particles. The culture was allowed to grow overnight at 37°C. The propagated phage was retrieved from the culture supernatant by precipitation with PEG 8000/NaCl. The second and third rounds of selection were performed automatically using King-Fisher programmable magnetic particle processor, which exposes the propagated phage to immobilized hPRL-R, followed by washing with TBS containing 0.1 % tween 20 (TBST). The propagated phage particles were also exposed to streptavidin magnetic beads with no immobilized target, followed by washing with TBST. Phage particles from washed “empty” beads and beads with immobilized hPRL-R were eluted using 100 mM DTT, and used to infect log-phase XL1-Blue *E. coli* cells. The number of colony forming units (CFU) was counted for “target” and compared with “no target” selection. The enrichment ratio was calculated as: (target specific CFU x dilution factor) /(random clones CFU x dilution factor). After the third round of selection the enrichment ratio was ~2,700, indicating the selection was successful.

**Characterization of clones by Phage enzyme-linked immunosorbent assay (ELISA)**

Individual clones from the third round of selection were isolated and subjected to a competitive phage ELISA to screen for hPRL-R specific binding. The competitive ELISA was performed on immobilized hPRL-R measuring the binding of the individual clones in the presence and absence of free hRPRL (competitor). Briefly, individual clones of phage in XL1-Blue *E. coli* were grown overnight in 300 μl of 2xYT in the presence of ampicillin (100μg/mL) and M13K07 helper phage (10^8^/mL) in 96-well plate format. Phage was isolated from the bacteria by centrifugation. A 96-well MaxiSorb NUNC plate (Fisher scientific) was coated for 1 hour with 2μg/ml Neutravidin (NAV) in coating buffer (0.1 Na2CO3, pH 9.6) and blocked overnight at 4°C with 0.5%BSA/TBS. Two wells were reserved per each clone; 50 μl of 20nM biotinylated hPRL-R was added to one of the wells, the other contained TBS. The plate was incubated for 30 min at room temperature and then washed five times with TBST. The culture supernatants containing phage clones were added at a three-fold dilution with 0.5% BSA/TBS (~10^7^ phage/μL). The plate was then incubated for 40 minutes at room temperature with gentle shaking and washed five times with TBST. The bound phage was probed with an HRP-linked anti-M13 antibody at a 1:2500 dilution (40 min incubation). The plate was washed with TBST and TBS, developed with Turbo TMB-ELISA substrate (Thermo) and quenched with 2 M H_2_SO_4_. Absorbance of each well was read at 450 nm on a BioTeK plate reader. Clones that exhibited signal at least five-fold higher than the signal from nonspecific binding (in the well without target protein) were identified as positive and considered for further analysis. Of the 20 colonies initially tested, 16 showed a positive ELISA competition test. The 16 clones were sequenced revealing 4 unique sequences, which were designated as sABs A4, A8, A9 and A10.

**Surface Plasmon Resonance**

Surface Plasmon resonance was performed on a BIAcore 2000 at 25ºC. The CM5 sensor chip (GE Healthcare) coupling was carried out in HBS-EP (10 mM HEPES, 150 mM NaCl, 3 mM EDTA, 0.005% TWEEN-20, pH 7.4) at a flow rate of 5 µl/min. Sensor chip was first activated by injection of 20μl of NHS/EDC (N-hydroxysuccinimide/N-ethy-N’-(3-dimethyl-amino-propyl)-carbodiimide, 75mg/ml:11.5mg/ml) mixture. The crosslinker 2-(2-Pyridinyldithio) ethaneamine hydrochloride (PDEA) (GE Healthcare) was suspended in 10 mM sodium acetate, pH 4.5 at 18mg/ml concentration and 10μl were injected. T207C hPRL-R was reduced prior to the experiment with 1.5 mM DTT to break apart the dimerized molecules. The protein was then suspended at 100μg/ml, 75μg/ml and 50μg/ml in sodium acetate buffer; 30μl of the samples was injected in three out of the four flow cells on the surface of the chip. Residual thiol groups of the PDEA were blocked with 50mM cysteine-HCl/1M NaCl. Finally, the chip was deactivated with 1M ethanolamine, pH 8.3. The analyzed sABs were injected for 1 minute at 50μl/min and their dissociation was observed for 6 minutes. The concentrations of injected sABs were 12.5 nM, 25 nM, 50 nM, 100 nM, 200 nM and 400 nM. Chips were regenerated with 20μl of 10mM glycine-HCl, pH 2.0. The obtained data were double referenced against the signal from the flow cell without immobilized receptor and that of buffer injections. The sensograms were analyzed using BIAEvaluation 3.0 software and fitted to a simple 1:1 Langmuir model (Supplementary Fig. 1). The kinetic parameters are shown in Supplementary Table 1.

**X-ray crystallography**

The purified complex of hPRL-R sAB-A8 was isolated on a size exclusion column and subjected to crystallization trials at 10mg/ml protein (total) concentration. Crystals were grown in 160 mM Calcium Acetate, 80 mM Cacodylate buffer, pH 6.2, 20% glycerol, 11% PEG 8,000. Crystals were dehydrated by successive soakings in higher glycerol versions of the crystal conditions. Data were collected at beam line 21-ID-D of LS-CAT at the Advanced Photon Source at Argonne National Laboratory. All data were integrated and scaled with the HKL2000 1.97 suite of programs. Phases were calculated from a molecular replacement solution identified using a sAB model (PDB: 2R8S) [4] and the N-terminal domain of the hPRLR from PDB 3D48 with Phaser [5] in CCP4 6.0 [6]. The structure has two sAB/hPRLR complexes per asymmetric unit and a solvent content of 52%. Model building was performed in Coot [7] and refinement was conducted with Refmac 5.4.0073 [8]. Restrained maximum-likelihood refinement was used with non-crystallographic symmetry restraints applied to identical domains. Data collection and refinement statistics are shown in Supplementary Table 1.

**Inhibition of hormone internalization**

The hPRL and hGH were labeled with cy5 in PBS pH 7.4 by addition of 5-fold molar excess of mono-reactive NHS-ester cy5 (GE Healthcare) to each protein, then incubated with end-over-end tumbling overnight at 4 °C. The labeled protein was isolated from excess dye using a DG-10 econopac gel-filtration column (Bio-Rad) equilibrated with PBS, pH 7.4. T47D cells were cultured in DMEM with 10% FBS (full medium). Cells were seeded on glass cover-slips in 12-well plates (~50,000/well) overnight in full medium, then starved in FBS-free, indicator-free RPMI overnight. Cells were pre-incubated with 2 µM sAB for 30 min, then 100 nM cy5-labeled PRL or hGH were added to the cells, incubated for 3 hrs, followed by fixing with 4% *p-*formaldehyde (20 min), washing with PBS, Hoechst (Invitrogen) nuclear staining (1:1000 dilution) and mounting on glass slides using Pro-Long Gold mounting reagent (Invitrogen). Samples treated with hGH were also treated with ZnCl_2_ (50 µM final concentration). The cells were imaged on a Zeiss Axio imager microscope. Images were merged in ImageJ.

**Inhibition of hPRL-R signaling**

The dual luciferase assay was carried out as described [9]. Briefly, T47D cells were double transfected in 12-well plates (~90,000 cells/well) with LHRE-pGL4 and Renilla pGL4.73 and incubated overnight in OPTI-MEM medium. The cells were starved in serum-free medium overnight, then hPRL (100 ng/mL) was added to the cells pre-incubated for 30 min with varying concentrations of each sAB in triplicates. After overnight incubation, the cells were washed with PBS, the luciferase activity for each sample was determined. The Dual-Glo Luciferase assay system (Promega) was carried out as described in the manufacturer’s protocol to determine the relative levels of luminescence of each sample. A sAB which binds to bacterial maltose binding protein (MOS1) [2] was used as a negative control as well as cells with no sAB added.

For analysis of the downstream phosphorylation signaling of hPRL-R, T47D cells were cultured and starved as described above. Cells were treated with 100 ng of hPRL in the presence or absence of 1 µM of each sAB, as well as the control sAB. The cells were lysed 0, 15 or 30 min after addition of hormone. Cell lysates from each sample were separated on an SDS polyacrylamide gel. Western blot was carried out using phospho-specific and non-phospho-specific antibodies against Stat5, Erk and Akt as described [10].

**Supplementary Table 1. Crystallographic parameters of the sAB A8-hPRLR complex**

| **Data collection** |  |
| --- | --- |
| Space group | P6_1_ |
| Cell dimensions   |  |
| *a*, *b*, *c* (Å) | 285.8, 285.8, 62.2 |
|     α, β, γ  (°) | 90, 90, 120 |
| Wavelength | 1.12714 |
| Resolution (Å) | 50-3.25 (3.37-3.25) |
| I/σI | 11.4 (1.9) |
| No. unique reflections | 47509 |
| *R*_sym_  (%) | 18.4 (69.2) |
| Completeness (%) | 99.4 (97.8) |
| Redundancy | 6.5 (4.3) |
|  |  |
| **Refinement** |  |
| Resolution (Å) | 50-3.25 |
| *R*_work_ / *R*_free_ | 19.4/24.6 |
| No. atoms |  |
| Protein | 9873 |
| Ion | 1 |
| R.m.s deviations |  |
| Bond lengths (Å) | 0.016 |
| Bond angles (°) | 2.1 |

**
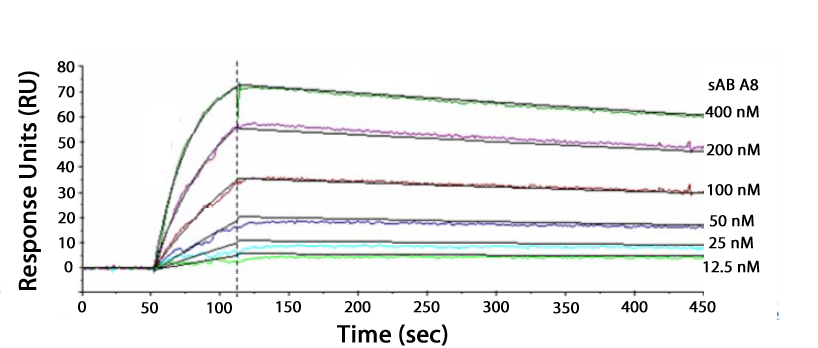
**

**Supplementary Fig 1.** Representative binding kinetics of sAB-hPRL-R interaction. SPR traces of various sAB A8 concentrations injected over a CM5 chip with immobilized hPRL-R (T207C). Response Units (RUs) are plotted on the Y-axis, and time (sec) is plotted on the X-axis. sAB is flowed over the chip for 2 minutes (up to dashed line), followed by buffer flow. The data were obtained on a Biacore 2000.

**
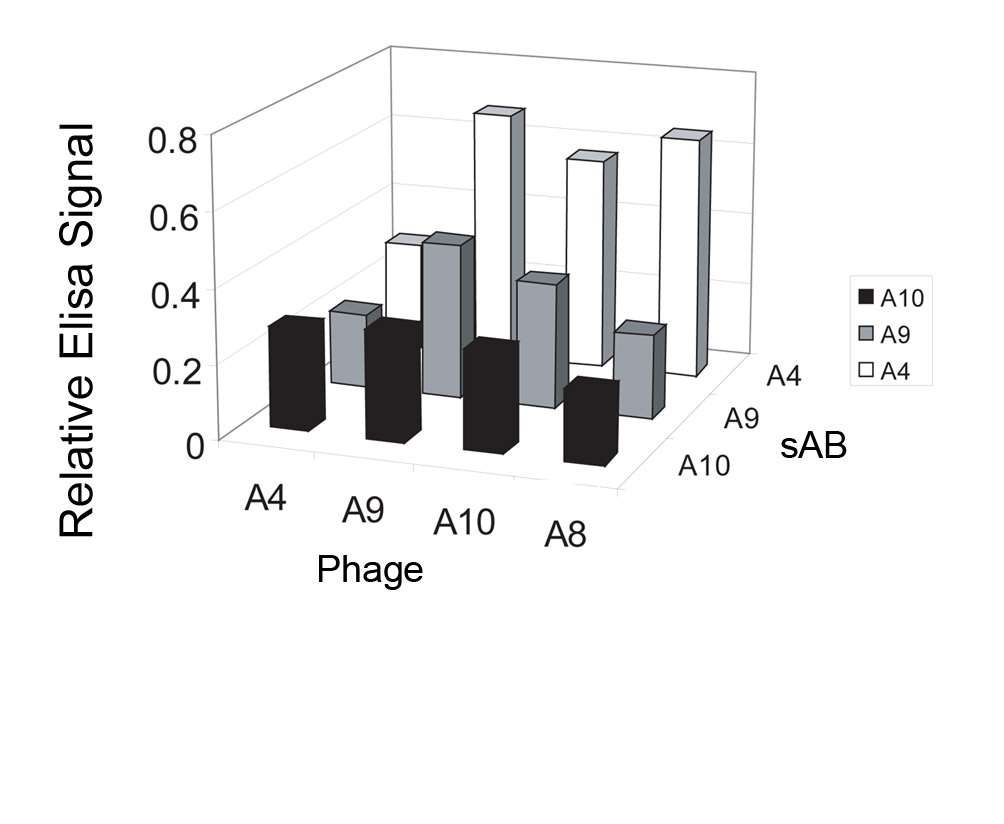

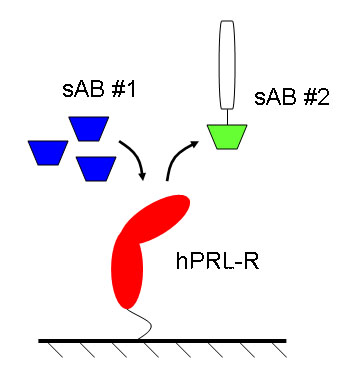
**

**Supplementary Fig 2.** Mapping the epitopes of sAB binding to hRPRL. Left panel: schematic of the concept. An immobilized hPRL-R T207C mutant (red) is incubated with phage displaying a sAB (green) in the presence of excess purified sAB (blue). Phage ELISA is used to determine the relative amount of bound phage, whereby a loss in ELISA signal indicates that sAB #1 interferes with binding of sAB #2 with hPRL-R. Right panel: phage ELISA epitope mapping data of intersecting sABs. Incubation of the receptor with sABs A9 and A10 interferes with binding of sABs A8, A9 and A10 (ELISA signal < 0.4 AU). The control sAB A4 does not interfere with binding of sABs A8, A9 or A10.

**References:**

1. Cunningham BC, Bass S, Fuh G, Wells JA: **Zinc mediation of the binding of human growth hormone to the human prolactin receptor.** *Science* 1990, **250:**1709-1712.

2. Rizk SS, Paduch M, Heithaus JH, Duguid EM, Sandstrom A, Kossiakoff AA: **Allosteric control of ligand-binding affinity using engineered conformation-specific effector proteins.** *Nat Struct Mol Biol* 2011, **18:**437-442.

3. Fellouse FA, Esaki K, Birtalan S, Raptis D, Cancasci VJ, Koide A, Jhurani P, Vasser M, Wiesmann C, Kossiakoff AA, et al: **High-throughput generation of synthetic antibodies from highly functional minimalist phage-displayed libraries.** *J Mol Biol* 2007, **373:**924-940.

4. Ye JD, Tereshko V, Frederiksen JK, Koide A, Fellouse FA, Sidhu SS, Koide S, Kossiakoff AA, Piccirilli JA: **Synthetic antibodies for specific recognition and crystallization of structured RNA.** *Proc Natl Acad Sci U S A* 2008, **105:**82-87.

5. Somers W, Ultsch M, De Vos AM, Kossiakoff AA: **The X-ray structure of a growth hormone-prolactin receptor complex.** *Nature* 1994, **372:**478-481.

6. Collaborative Computational Project n: **The CCP4 suite: programs for protein crystallography.** *Acta Crystallogr D Biol Crystallogr* 1994, **50:**760-763.

7. Emsley P, Lohkamp B, Scott WG, Cowtan K: **Features and development of Coot.** *Acta Crystallogr D Biol Crystallogr* 2010, **66:**486-501.

8. Murshudov GN, Vagin AA, Dodson EJ: **Refinement of macromolecular structures by the maximum-likelihood method.** *Acta Crystallogr D Biol Crystallogr* 1997, **53:**240-255.

9. Fang F, Antico G, Zheng J, Clevenger CV: **Quantification of PRL/Stat5 signaling with a novel pGL4-CISH reporter.** *BMC Biotechnol* 2008, **8:**11.

10. Zheng J, Fang F, Zeng X, Medler TR, Fiorillo AA, Clevenger CV: **Negative cross talk between NFAT1 and Stat5 signaling in breast cancer.** *Mol Endocrinol* 2011, **25:**2054-2064.
